# Supplementary material for: Mutation in the mitochondrial chaperone TRAP1 leads to autism with more severe symptoms in males
Source: EMBO Mol Med. 2024 Sep 27;16(11):2976–3004. doi: 10.1038/s44321-024-00147-6 (PMC11554806; doi:10.1038/s44321-024-00147-6)
Supplement: Supplementary file 1 — Appendix [file 44321_2024_147_MOESM1_ESM.pdf]

Appendix

Mutation in the mitochondrial chaperone TRAP1 leads to autism with more severe symptoms in males.

Authors: Małgorzata Rydzanicz[1]†, Bozena Kuzniewska[2,3]†, Marta Magnowska[2,3], Tomasz Wójtowicz[4], Aleksandra Stawikowska[2,3], Anna Hojka[5], Ewa Borsuk[6], Ksenia Meyza[7], Olga Gewartowska[8], Jakub Gruchota[9], Jacek Miłek[2,3], Patrycja Wardaszka[2], Izabela Chojnicka[10], Ludwika Kondrakiewicz[7], Dorota Dymkowska[11], Alicja Puścian[7], Ewelina Knapska[7], Andrzej Dziembowski[9,6]*, Rafał Płoski[1]* and Magdalena Dziembowska[2,3]*

Affiliations:

[1]Department of Medical Genetics, Medical University of Warsaw, Warsaw, Poland.
[2]Department of Physiology, Faculty of Biology, University of Warsaw, Warsaw, Poland
[3] Centre of New Technologies, University of Warsaw; Banacha 2c, Warsaw, Poland
[4] Laboratory of Cell Biophysics, Nencki Institute of Experimental Biology, Warsaw, Poland
[5] Bioinformatics Core Facility, International Institute of Molecular and Cell Biology in Warsaw, Poland
[6] Department of Embryology, Faculty of Biology, University of Warsaw, Warsaw Poland
[7]Laboratory of Emotions Neurobiology, Nencki Institute of Experimental Biology, Warsaw, Poland
[8] Genome Engineering Facility, International Institute of Molecular and Cell Biology in Warsaw, Poland
[9]Laboratory of RNA Biology, International Institute of Molecular and Cell Biology in Warsaw, Poland
[10]Department of Health and Rehabilitation Psychology, Faculty of Psychology, University of Warsaw, Warsaw, Poland
[11]Laboratory of Cellular Metabolism, Nencki Institute of Experimental Biology, Warsaw, Poland

† These authors contributed equally to this work

*Corresponding authors. Emails: m.dziembowska@cent.uw.edu.pl
rafal.ploski@wum.edu.pl
adziembowski@iimcb.gov.pl

Table of contents

Appendix Figure S1, related to Figure 2 ….…………………………………………….3

Appendix Figure S2, related to Figure 2 ….…………………………………………….4

Appendix Figure S3, related to Figure 2 ……………………………………………...5

Appendix Figure S4, related to Figure 4 ...……………………………………………7

Appendix Table S1, list of statistical method used and p-values .....…………………..…9

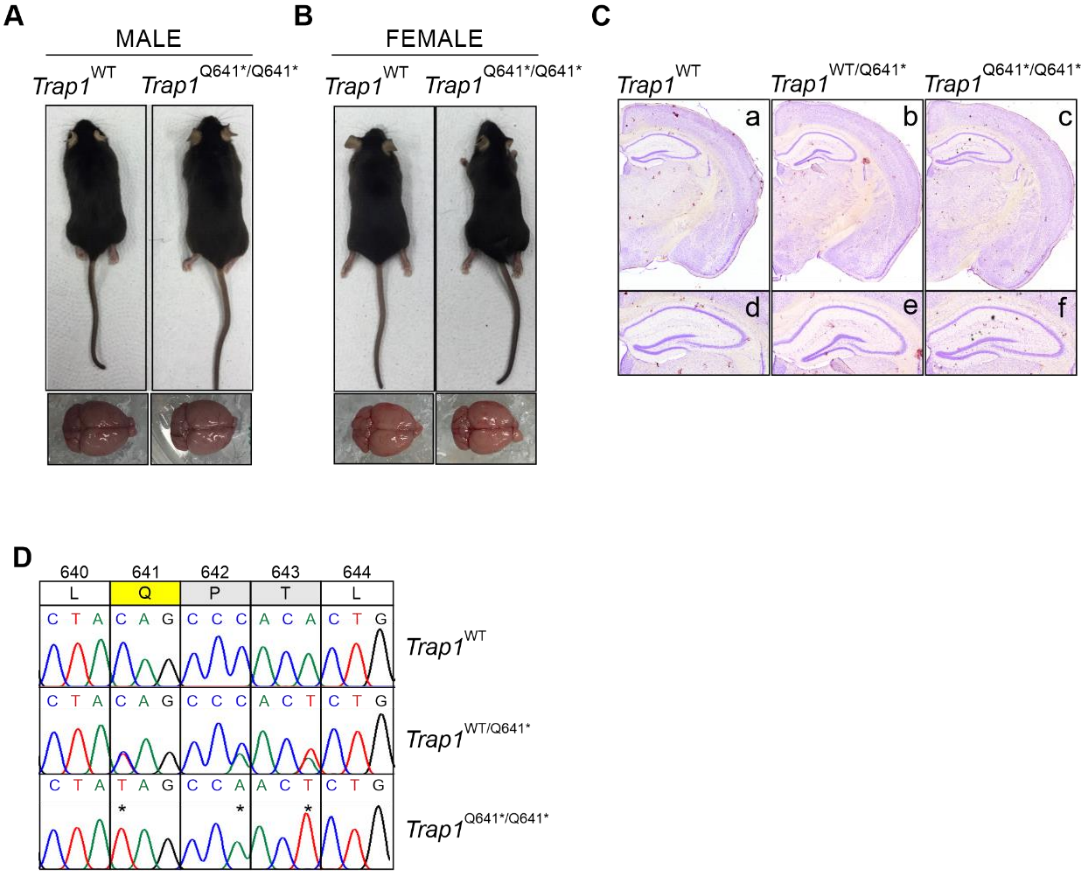

Appendix Figure S1, related to Figure 2

Phenotypic characterization of adult *Trap1*^{WT} and *Trap1*$^{Q641*/Q641*}$ mice.

(A, B) Photos of *Trap1*^{WT} and *Trap1*$^{Q641*/Q641*}$ male and female mice at 12 weeks of age. No clear aberrant phenotype was observed in *Trap1*$^{Q641*/Q641*}$ mice. No gross anatomical differences in the brains of *Trap1*^{Q641*} mice were observed. **(C)** Nissl-stained coronal sections of brains from wild type **(a, d)** heterozygous **(b, e)** and homozygous mutant **(c, f)** mice. No gross neuroanatomical differences were observed. **(D)** Alignment of chromatograms covering mutation site in *Trap1*: wild-type (*Trap1*^{WT}, top), heterozygous (*Trap1*$^{WT/Q641*}$, middle) and mutant (*Trap1*^{Q641*}, bottom). Mutated nt are marked with asterisks.

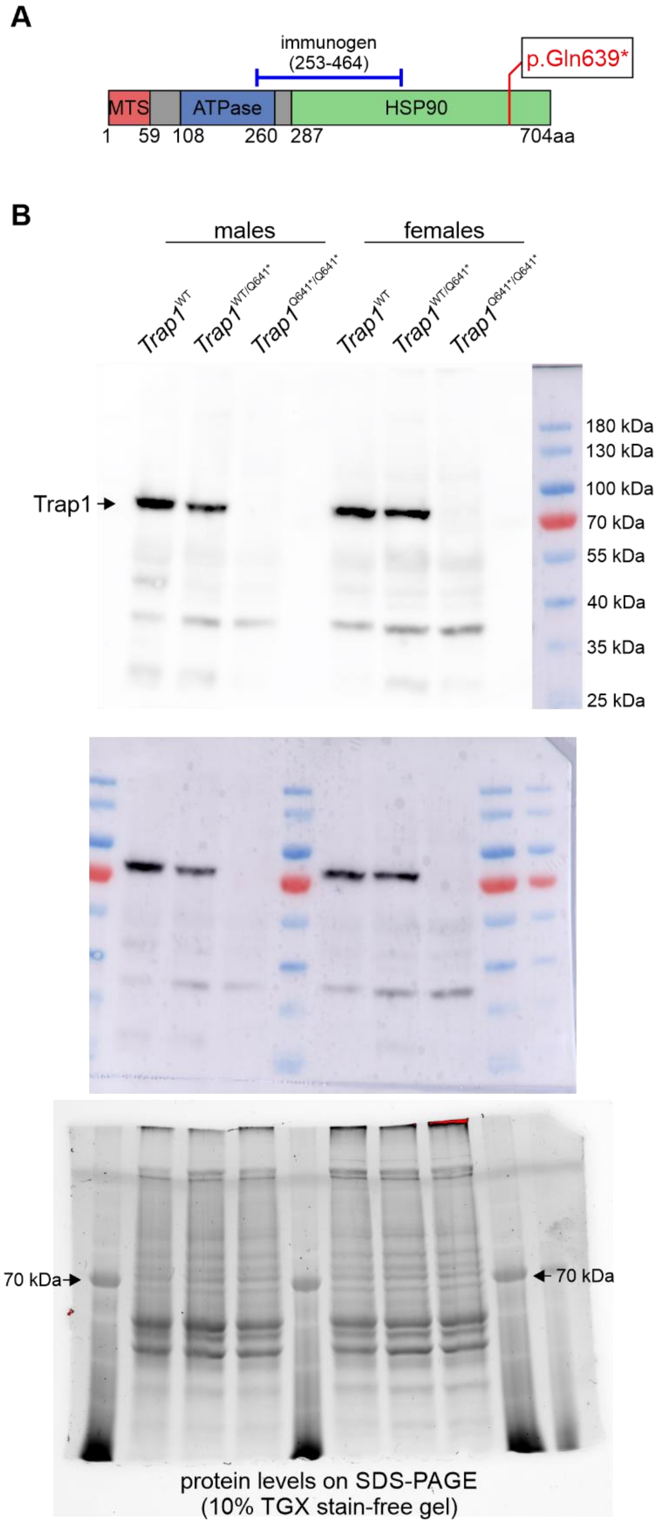

Appendix Figure S2, related to Figure 2

(A) Schematic representation of domain organization of TRAP1 protein. The mutation site and the immunogenic region detected by the used antibody are shown. (B) Full-size anti-Trap1 western blot image, that shows that no signal for truncated Trap1 p.Q639 protein was detected. Bottom panel shows the protein loading on the SDS-PAGE TGX-stain free gel.

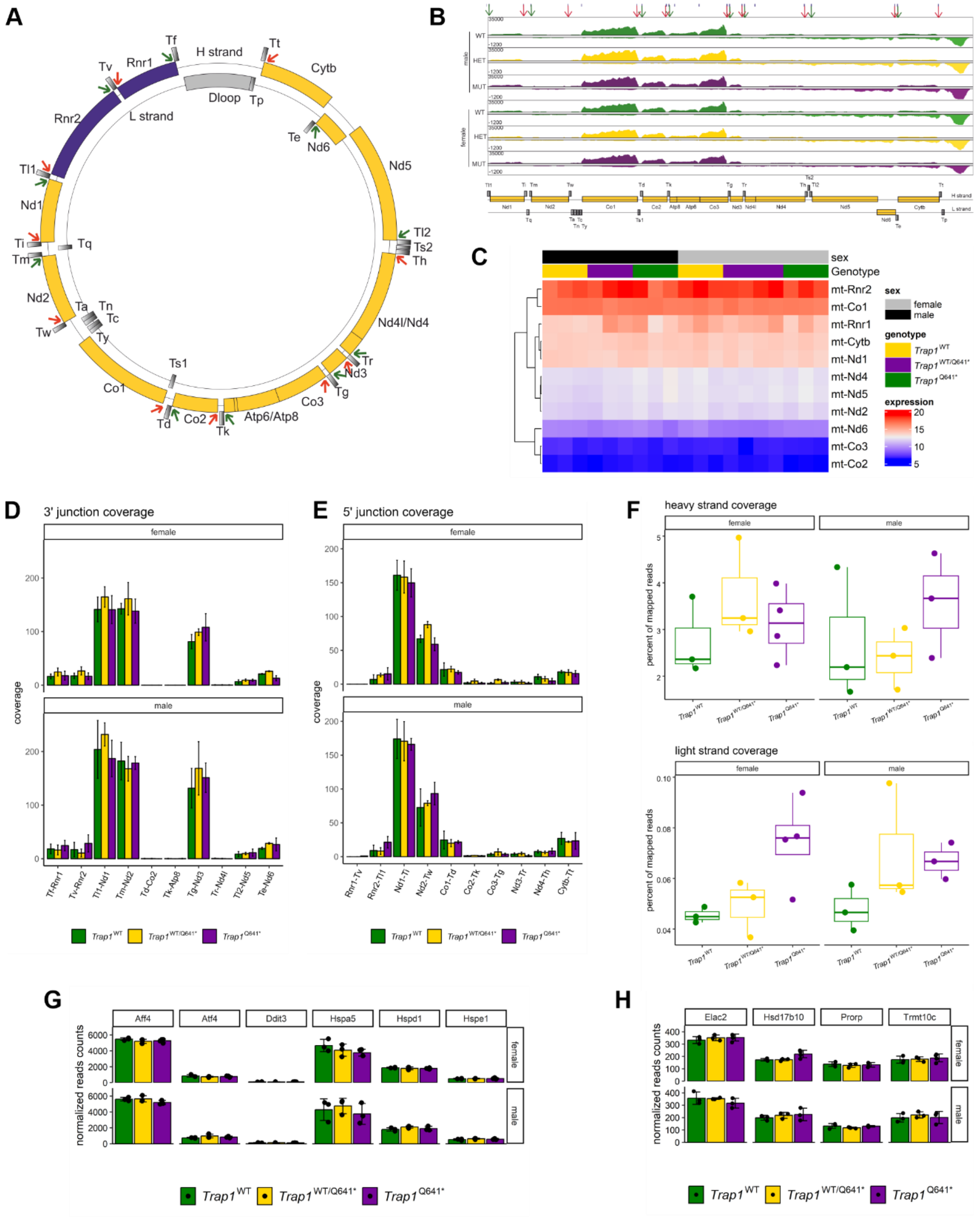

Appendix Figure S3, related to Figure 2

RNA Sequencing analysis of the hippocampi of *Trap1*^{WT}, *Trap1*$^{WT/Q641*}$ and *Trap1*$^{Q641*/Q641*}$ mice

(A). Map of the mitochondrial genome. The H (heavy, outer circle) and L (light, inner circle) strands are shown with their corresponding genes. The green and red arrows indicate the 5` and 3` junctions, respectively, that were analyzed for pre-RNA processing dysregulation. **(B)** Visualization of alignment of RNA-Seq reads mapped to the mitochondrial genome. Screenshots of the per base coverage visualized with the IGV v2.3 viewer. **(C)** Heatmap

representation of expression of genes encoded by the mitochondrial genome on transcript levels from the RNA-Seq analysis. The colors represent the vsd normalized reads. Blue represents low expression, whereas red represents high expression. **(D, E)** Analysis of mitochondrial pre-RNA processing defects based on the number of reads crossing the tRNA/mRNA gene junction. The reads were calculated by the number that map on the crossing junction assigned at 10 nt upstream and downstream of the real 1-nt junction. **(F)** The percentage contribution of mitochondrial reads in total mapped RNA-Seq reads for the H and L strands. **(G, H)** Levels of transcripts in the RNA-seq data encoding proteins involved in the unfolded protein response (G) and in mitochondrial tRNA processing (H).

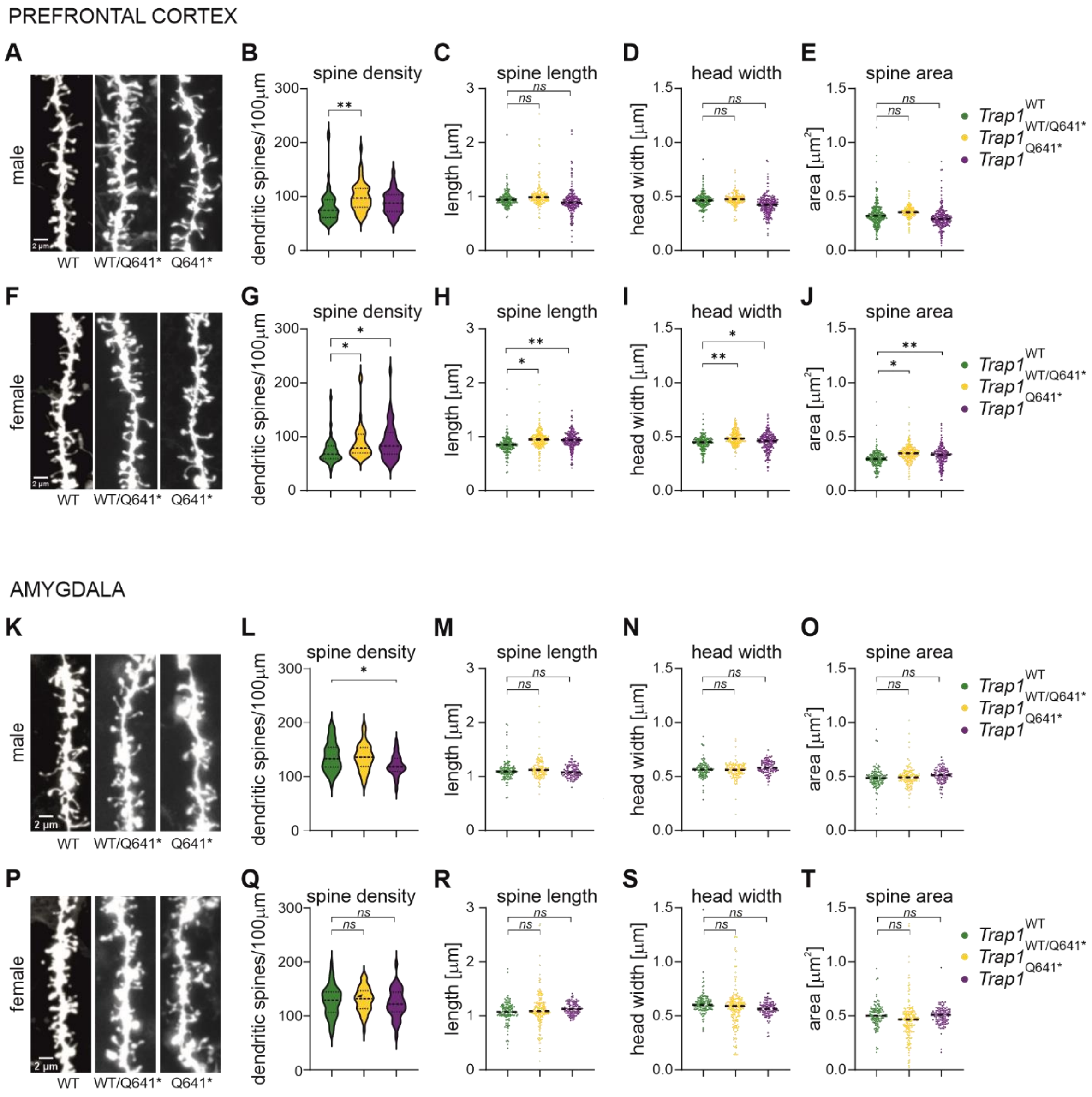

Appendix Figure S4, related to Figure 4

Morphometric analysis of dendritic spines in the medial prefrontal cortex and amygdala of *Trap1*^{WT}, *Trap1*$^{WT/Q641*}$ and *Trap1*$^{Q641*/Q641*}$ mice

(A, F, K, P) Representative images of DiI stained dendrites in the medial prefrontal cortex (mPFC) **(A, F)** and amygdala **(K, P)** of male and female *Trap1* mice. Scale bars 2 µm. **(B, G)** Mean density of dendritic spines in mPFC. Plot shows mean density/100 µm of dendrite; n=35-53 (males), n=41-44 (females) images/group; *p<0.05, **p<0.01; one way ANOVA, *post-hoc* Tukey's test. **(C-E, H-J)**. Dendritic spine morphology in *Trap1* male (C-E) and female (H-J) medial prefrontal cortex. Plots show mean value for spine length, spine head width, and spine area; n= 3727-6746 (males), 4350-5500 (females) analyzed spines/experimental group; *p<0.05, **p<0.01,***p<0.001; nested ANOVA, *post-hoc* Tukey's test. N=3-6 animals/group (males), N=3-4 animals/group (females). **(L, Q)** Mean density of dendritic spines in the amygdala. Plot shows mean density/100 µm of dendrite; n=34-65 (males), n=32-52 (females) images/group; *p<0.05, **p<0.01; one way ANOVA, *post-hoc* Tukey's test. **(M-O, R-T)**

Dendritic spine morphology in *Trap1* male and female amygdala. Plots show mean value for spine length, spine head width, and spine area; n= 2496-4760 (males), 2695-4184 (females) analyzed spines/experimental group; *p<0.05, **p<0.01,***p<0.001; nested ANOVA, *post-hoc* Tukey's test. N=3-6 animals/group (males), N=3-5 animals/group (females).

Appendix Table S1

Figure Number	P Value	Method	
Figure 2C	<0,0001	One-way ANOVA	***
F WT vs. F het	<0,0001	Sidak's multiple comparisons test	***
F WT vs. F MUT	<0,0001	Sidak's multiple comparisons test	***
F WT vs. M WT	0,2522	Sidak's multiple comparisons test	ns
M WT vs. M het	<0,0001	Sidak's multiple comparisons test	***
M WT vs. M MUT	<0,0001	Sidak's multiple comparisons test	***
F MUT vs. M MUT	>0,9999	Sidak's multiple comparisons test	ns
Figure 2D	<0,0001	One-way ANOVA	***
M WT vs. M het	<0,0001	Sidak's multiple comparisons test	***
M WT vs. M MUT	<0,0001	Sidak's multiple comparisons test	***
F WT vs. F het	0,0002	Sidak's multiple comparisons test	***
F WT vs. F MUT	<0,0001	Sidak's multiple comparisons test	***
Figure 3D			
M WT vs. M het	<0,0001	Kolmogorov-Smirnov test	***
M WT vs. M MUT	0,0011	Kolmogorov-Smirnov test	**
Figure 3E			
F WT vs. F het	0,0016	Kolmogorov-Smirnov test	**
F WT vs. F MUT	0,0532	Kolmogorov-Smirnov test	ns
Figure 3G		Two-way ANOVA	
WT (social vs. non-social)	0,0101	Uncorrected Fisher's LSD	*
Q641*/Q641* (social vs. non-social)	0,9991	Uncorrected Fisher's LSD	ns
social (WT vs. Q641*/Q641*)	0,0409	Uncorrected Fisher's LSD	*
non-social (WT vs. Q641*/Q641*)	0,6994	Uncorrected Fisher's LSD	ns
Figure 3H		Two-way ANOVA	
WT (social vs. non-social)	0,0195	Uncorrected Fisher's LSD	*
Q641*/Q641* (social vs. non-social)	0,3385	Uncorrected Fisher's LSD	ns
social (WT vs. Q641*/Q641*)	0,0304	Uncorrected Fisher's LSD	*
non-social (WT vs. Q641*/Q641*)	0,4375	Uncorrected Fisher's LSD	ns
Figure 4B	0,0024	One-way ANOVA	**
WT vs. HET	0,0015	Tukey's multiple comparisons test	**
WT vs. MUT	0,1789	Tukey's multiple comparisons test	ns
HET vs. MUT	0,1218	Tukey's multiple comparisons test	ns
Figure 4C	0,0004	Nested one-way ANOVA	***
WT vs. HET	0,1277	Tukey's multiple comparisons test	ns
WT vs. MUT	0,0335	Tukey's multiple comparisons test	*
HET vs. MUT	0,0002	Tukey's multiple comparisons test	***
Figure 4D	<0,0001	Nested one-way ANOVA	***
WT vs. HET	0,9254	Tukey's multiple comparisons test	ns
WT vs. MUT	0,0002	Tukey's multiple comparisons test	***
HET vs. MUT	0,0002	Tukey's multiple comparisons test	***
Figure 4E	<0,0001	Nested one-way ANOVA	***
WT vs. HET	0,472	Tukey's multiple comparisons test	ns
WT vs. MUT	0,0006	Tukey's multiple comparisons test	***
HET vs. MUT	<0,0001	Tukey's multiple comparisons test	***
Figure 4G	0,0149	One-way ANOVA	*
WT vs. HET	0,7141	Tukey's multiple comparisons test	ns
WT vs. MUT	0,0137	Tukey's multiple comparisons test	*
HET vs. MUT	0,1436	Tukey's multiple comparisons test	ns
Figure 4H	0,0006	Nested one-way ANOVA	***

WT vs. HET	0,1598	Tukey's multiple comparisons test	ns
WT vs. MUT	0,0003	Tukey's multiple comparisons test	***
HET vs. MUT	0,1639	Tukey's multiple comparisons test	ns
Figure 4I	0,003	Nested one-way ANOVA	**
WT vs. HET	0,1925	Tukey's multiple comparisons test	ns
WT vs. MUT	0,0019	Tukey's multiple comparisons test	**
HET vs. MUT	0,3116	Tukey's multiple comparisons test	ns
Figure 4J	0,0003	Nested one-way ANOVA	***
WT vs. HET	0,1303	Tukey's multiple comparisons test	ns
WT vs. MUT	0,0002	Tukey's multiple comparisons test	***
HET vs. MUT	0,149	Tukey's multiple comparisons test	ns
Figure 4K			
Males (fEPSP slope)	0,0003	Kruskal-Wallis test	***
M WT vs. M HET	0,8665	Dunn's multiple comparisons test	ns
M WT vs. M MUT	0,0135	Dunn's multiple comparisons test	*
M HET vs. M MUT	0,0003	Dunn's multiple comparisons test	***
Figure 4L			
Males (fEPSP area)	0,0081	Kruskal-Wallis test	**
M WT vs. M HET	0,2377	Dunn's multiple comparisons test	ns
M WT vs. M MUT	0,0059	Dunn's multiple comparisons test	**
M HET vs. M MUT	0,5422	Dunn's multiple comparisons test	ns
Figure 4M			
Males (paired-pulse ratio data)	0,0018	Two-Way RM ANOVA	**
25ms Inter Stimulus Interval			
(paired-pulse ratio)	0,201	Tukey's multiple comparisons test	ns
(paired-pulse ratio)	0,0142	Tukey's multiple comparisons test	*
(paired-pulse ratio)	0,3694	Tukey's multiple comparisons test	ns
50ms Inter Stimulus Interval			
M WT vs. M HET (paired-pulse ratio)	0,1566	Tukey's multiple comparisons test	ns
M WT vs. M MUT (paired-pulse ratio)	0,0043	Tukey's multiple comparisons test	**
M HET vs. M MUT (paired-pulse ratio)	0,367	Tukey's multiple comparisons test	ns
100ms Inter Stimulus Interval			
M WT vs. M HET (paired-pulse ratio)	0,8271	Tukey's multiple comparisons test	ns
M WT vs. M MUT (paired-pulse ratio)	0,0551	Tukey's multiple comparisons test	ns
M HET vs. M MUT (paired-pulse ratio)	0,1687	Tukey's multiple comparisons test	ns
200ms Inter Stimulus Interval			
M WT vs. M HET (paired-pulse ratio)	0,9982	Tukey's multiple comparisons test	ns
M WT vs. M MUT (paired-pulse ratio)	0,3053	Tukey's multiple comparisons test	ns
M HET vs. M MUT (paired-pulse ratio)	0,1903	Tukey's multiple comparisons test	ns
Figure 4N			
Females (fEPSP slope)	0,0068	Kruskal-Wallis test	***
F WT vs. F HET	0,2719	Dunn's multiple comparisons test	ns
F WT vs. F MUT	0,0048	Dunn's multiple comparisons test	**
F HET vs. F MUT	0,429	Dunn's multiple comparisons test	ns
Figure 4O			
Females (fEPSP area)	0,0108	Kruskal-Wallis test	*
F WT vs. F HET	0,0565	Dunn's multiple comparisons test	ns
F WT vs. F MUT	0,0152	Dunn's multiple comparisons test	*
F HET vs. F MUT	>0,9999	Dunn's multiple comparisons test	ns
Figure 4P			
Females (paired-pulse ratio data)	0,8276	Two-Way RM ANOVA	**
25ms Inter Stimulus Interval			
F WT vs. F HET (paired-pulse ratio)	0,976	Tukey's multiple comparisons test	ns
F WT vs. F MUT (paired-pulse ratio)	0,9947	Tukey's multiple comparisons test	ns
F HET vs. F MUT (paired-pulse ratio)	0,8606	Tukey's multiple comparisons test	ns
50ms Inter Stimulus Interval			

F WT vs. F HET (paired-pulse ratio)	0,795	Tukey's multiple comparisons test	ns
F WT vs. F MUT (paired-pulse ratio)	0,8958	Tukey's multiple comparisons test	ns
F HET vs. F MUT (paired-pulse ratio)	0,9222	Tukey's multiple comparisons test	ns
100ms Inter Stimulus Interval			
F WT vs. F HET (paired-pulse ratio)	0,9388	Tukey's multiple comparisons test	ns
F WT vs. F MUT (paired-pulse ratio)	0,9931	Tukey's multiple comparisons test	ns
F HET vs. F MUT (paired-pulse ratio)	0,8833	Tukey's multiple comparisons test	ns
200ms Inter Stimulus Interval			
F WT vs. F HET (paired-pulse ratio)	0,8857	Tukey's multiple comparisons test	ns
F WT vs. F MUT (paired-pulse ratio)	0,858	Tukey's multiple comparisons test	ns
F HET vs. F MUT (paired-pulse ratio)	0,9837	Tukey's multiple comparisons test	ns
Figure 5G		One-way ANOVA	
HET vs. MUT	0,024	Tukey's multiple comparisons test	*
HET vs. WT	0,0052	Tukey's multiple comparisons test	**
MUT vs. WT	0,0001	Tukey's multiple comparisons test	***
Figure 5H		nested ANOVA	
HET vs. MUT	0,6603	Tukey's multiple comparisons test	ns
HET vs. WT	0,2337	Tukey's multiple comparisons test	ns
MUT vs. WT	0,0916	Tukey's multiple comparisons test	ns
Figure 5I		nested ANOVA	
HET vs. MUT	0,4661	Tukey's multiple comparisons test	ns
HET vs. WT	0,4539	Tukey's multiple comparisons test	ns
MUT vs. WT	0,104	Tukey's multiple comparisons test	ns
Figure 5K			
M MUT vs. M WT (Complex I-IV)	0,0256	Ratio paired t test	*
M MUT vs. M WT (Complex II-IV)	0,4676	Ratio paired t test	ns
M MUT vs. M WT (Complex IV)	0,0567	Ratio paired t test	ns
Figure 5L			
F MUT vs. F WT (Complex I-IV)	0,0279	Ratio paired t test	*
F MUT vs. F WT (Complex II-IV)	0,1899	Ratio paired t test	ns
F MUT vs. F WT (Complex IV)	0,9956	Ratio paired t test	ns
Figure EV5C		Two-Way RM ANOVA	
a-D-Glucose			
WT vs. het	0,9093	Dunnett's multiple comparisons test	ns
WT vs. MUT	0,9862	Dunnett's multiple comparisons test	ns
Glycogen			
WT vs. het	0,9489	Dunnett's multiple comparisons test	ns
WT vs. MUT	0,8649	Dunnett's multiple comparisons test	ns
D-Glucose-1-PO4			
WT vs. het	0,438	Dunnett's multiple comparisons test	ns
WT vs. MUT	0,9241	Dunnett's multiple comparisons test	ns
D-Glucose-6-PO4			
WT vs. het	0,7803	Dunnett's multiple comparisons test	ns
WT vs. MUT	0,9335	Dunnett's multiple comparisons test	ns
D-Gluconate-6-PO4			
WT vs. het	0,9646	Dunnett's multiple comparisons test	ns
WT vs. MUT	0,9752	Dunnett's multiple comparisons test	ns
D,L-a-Glycerol-PO4			
WT vs. het	0,8175	Dunnett's multiple comparisons test	ns
WT vs. MUT	0,1264	Dunnett's multiple comparisons test	ns
L-Lactic Acid			
WT vs. het	0,939	Dunnett's multiple comparisons test	ns
WT vs. MUT	0,9421	Dunnett's multiple comparisons test	ns
Pyruvic Acid			
WT vs. het	0,7393	Dunnett's multiple comparisons test	ns
WT vs. MUT	0,8037	Dunnett's multiple comparisons test	ns
Citric Acid			
WT vs. het	0,7518	Dunnett's multiple comparisons test	ns

WT vs. MUT	0,9867	Dunnett's multiple comparisons test	ns
D,L-Isocitric Acid			
WT vs. het	0,8137	Dunnett's multiple comparisons test	ns
WT vs. MUT	0,4772	Dunnett's multiple comparisons test	ns
cis-Aconitic Acid			
WT vs. het	0,985	Dunnett's multiple comparisons test	ns
WT vs. MUT	0,7274	Dunnett's multiple comparisons test	ns
a-Keto-Glutaric Acid			
WT vs. het	0,8487	Dunnett's multiple comparisons test	ns
WT vs. MUT	0,1239	Dunnett's multiple comparisons test	ns
Succinic Acid			
WT vs. het	0,4904	Dunnett's multiple comparisons test	ns
WT vs. MUT	<0,0001	Dunnett's multiple comparisons test	***
Fumaric Acid			
WT vs. het	0,0091	Dunnett's multiple comparisons test	**
WT vs. MUT	0,2005	Dunnett's multiple comparisons test	ns
L-Malic Acid			
WT vs. het	0,9939	Dunnett's multiple comparisons test	ns
WT vs. MUT	0,5085	Dunnett's multiple comparisons test	ns
L-Malic Acid 100uM			
WT vs. het	0,6394	Dunnett's multiple comparisons test	ns
WT vs. MUT	0,1812	Dunnett's multiple comparisons test	ns
Pyruvic Acid + L-Malic Acid 100uM			
WT vs. het	0,738	Dunnett's multiple comparisons test	ns
WT vs. MUT	0,016	Dunnett's multiple comparisons test	*
a-Keto-Butyric Acid			
WT vs. het	0,9449	Dunnett's multiple comparisons test	ns
WT vs. MUT	0,8074	Dunnett's multiple comparisons test	ns
D,L-ß-Hydroxy-Butyric Acid			
WT vs. het	0,8036	Dunnett's multiple comparisons test	ns
WT vs. MUT	0,0785	Dunnett's multiple comparisons test	ns
Acetyl-L-Carnitine + L-Malic Acid 100uM			
WT vs. het	0,6164	Dunnett's multiple comparisons test	ns
WT vs. MUT	0,9938	Dunnett's multiple comparisons test	ns
Octanoyl-L-Carnitine + L-Malic Acid 100uM			
WT vs. het	0,9728	Dunnett's multiple comparisons test	ns
WT vs. MUT	0,7963	Dunnett's multiple comparisons test	ns
Palmitoyl-D,L-Carnitine + L-Malic Acid 100uM			
WT vs. het	0,797	Dunnett's multiple comparisons test	ns
WT vs. MUT	0,9955	Dunnett's multiple comparisons test	ns
a-Keto-Isocaproic Acid + L-Malic Acid 100uM			
WT vs. het	0,5923	Dunnett's multiple comparisons test	ns
WT vs. MUT	0,9991	Dunnett's multiple comparisons test	ns
L-Glutamic Acid			
WT vs. het	0,2935	Dunnett's multiple comparisons test	ns
WT vs. MUT	0,5162	Dunnett's multiple comparisons test	ns
L-Glutamine			
WT vs. het	0,1423	Dunnett's multiple comparisons test	ns
WT vs. MUT	0,6386	Dunnett's multiple comparisons test	ns
Ala-Gln			
WT vs. het	0,357	Dunnett's multiple comparisons test	ns
WT vs. MUT	0,347	Dunnett's multiple comparisons test	ns
L-Serine			
WT vs. het	0,6513	Dunnett's multiple comparisons test	ns
WT vs. MUT	0,6462	Dunnett's multiple comparisons test	ns
L-Ornithine			
WT vs. het	0,8035	Dunnett's multiple comparisons test	ns
WT vs. MUT	0,8033	Dunnett's multiple comparisons test	ns
Tryptamine			
WT vs. het	0,4634	Dunnett's multiple comparisons test	ns
WT vs. MUT	0,9418	Dunnett's multiple comparisons test	ns
a-Amino-Butyric Acid + L-Malic Acid 100uM			
WT vs. het	0,7483	Dunnett's multiple comparisons test	ns
WT vs. MUT	0,992	Dunnett's multiple comparisons test	ns

L-Leucine + L-Malic Acid 100uM

WT vs. het	0,6313	Dunnett's multiple comparisons test	ns
WT vs. MUT	0,9993	Dunnett's multiple comparisons test	ns

legend:	
female WT	F WT
female WT/Q641*	F HET
female Q641*/Q641*	F MUT
male WT	M WT
male WT/Q641*	M HET
male Q641*/Q641*	M MUT
