# Supplementary figures and images for: Mutation in the mitochondrial chaperone TRAP1 leads to autism with more severe symptoms in males
Source: EMBO Mol Med. 2024 Sep 27;16(11):2976–3004. doi: 10.1038/s44321-024-00147-6 (PMC11554806; doi:10.1038/s44321-024-00147-6)

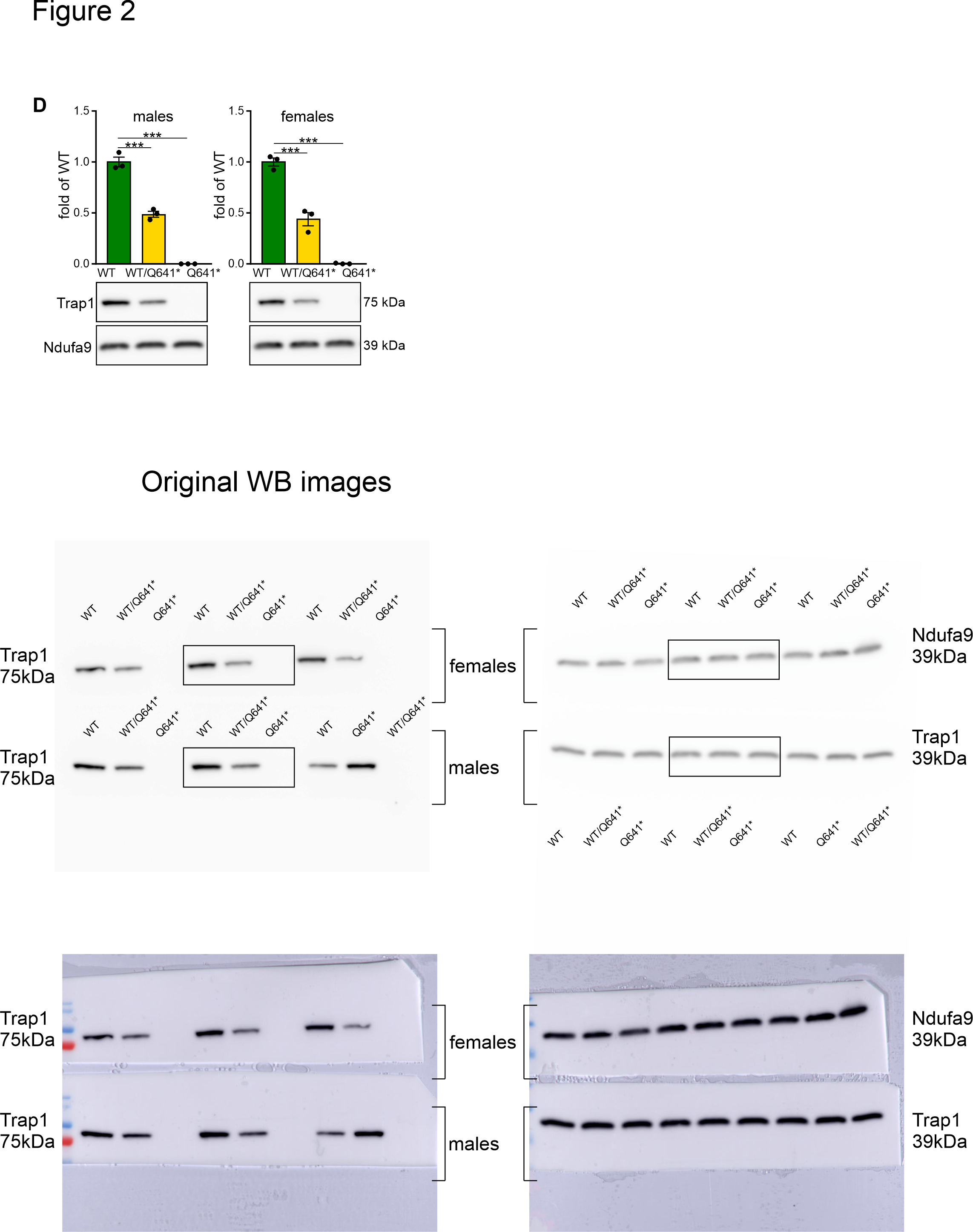

Supplement: Supplementary file 4 — Source data Fig. 2 [file 44321_2024_147_MOESM4_ESM.zip › Figure 2/2D/western blot Trap1.tif]

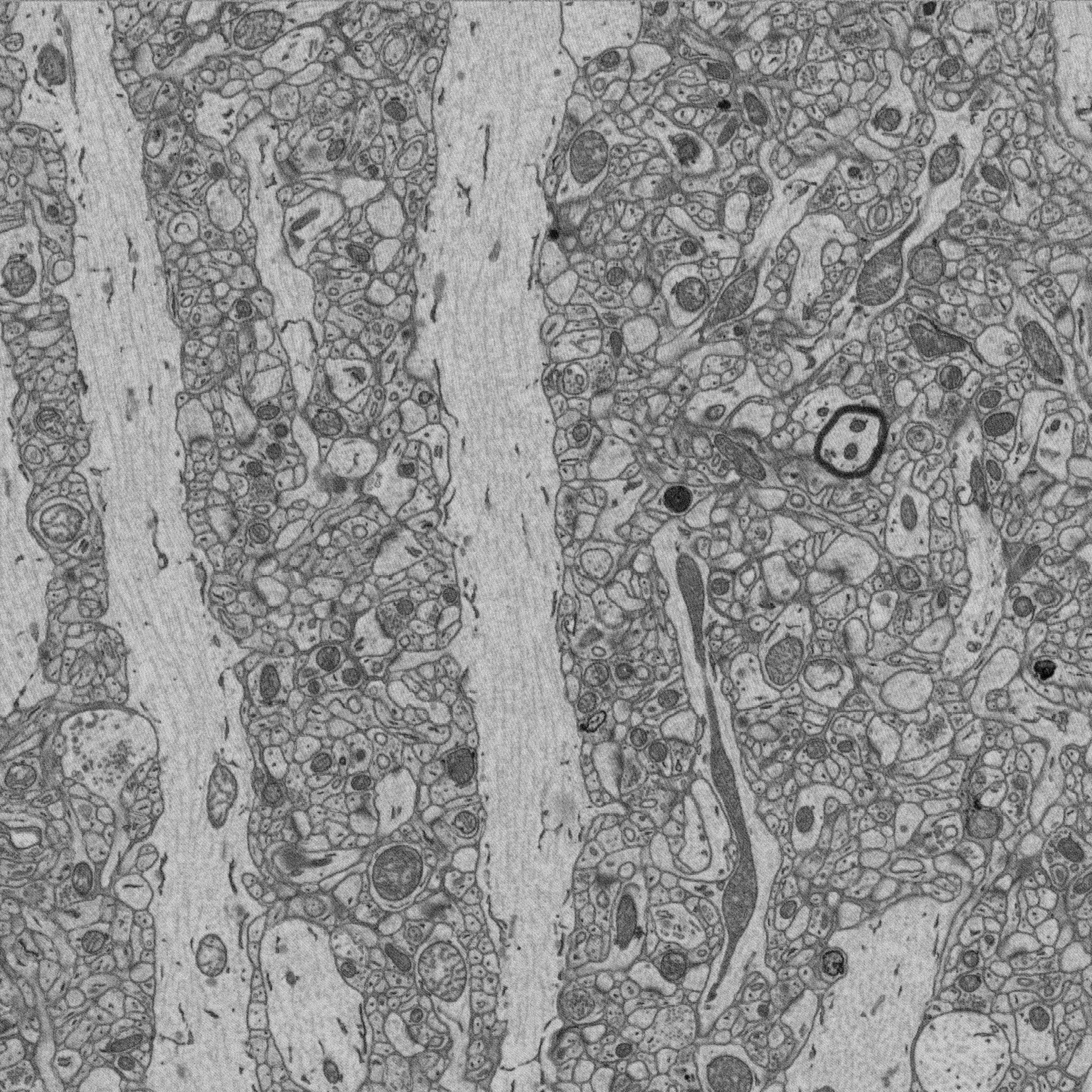

Supplement: Supplementary file 7 — Source data Fig. 5 [file 44321_2024_147_MOESM7_ESM.zip › Figure 5/5D/Figure 5 D.tif]

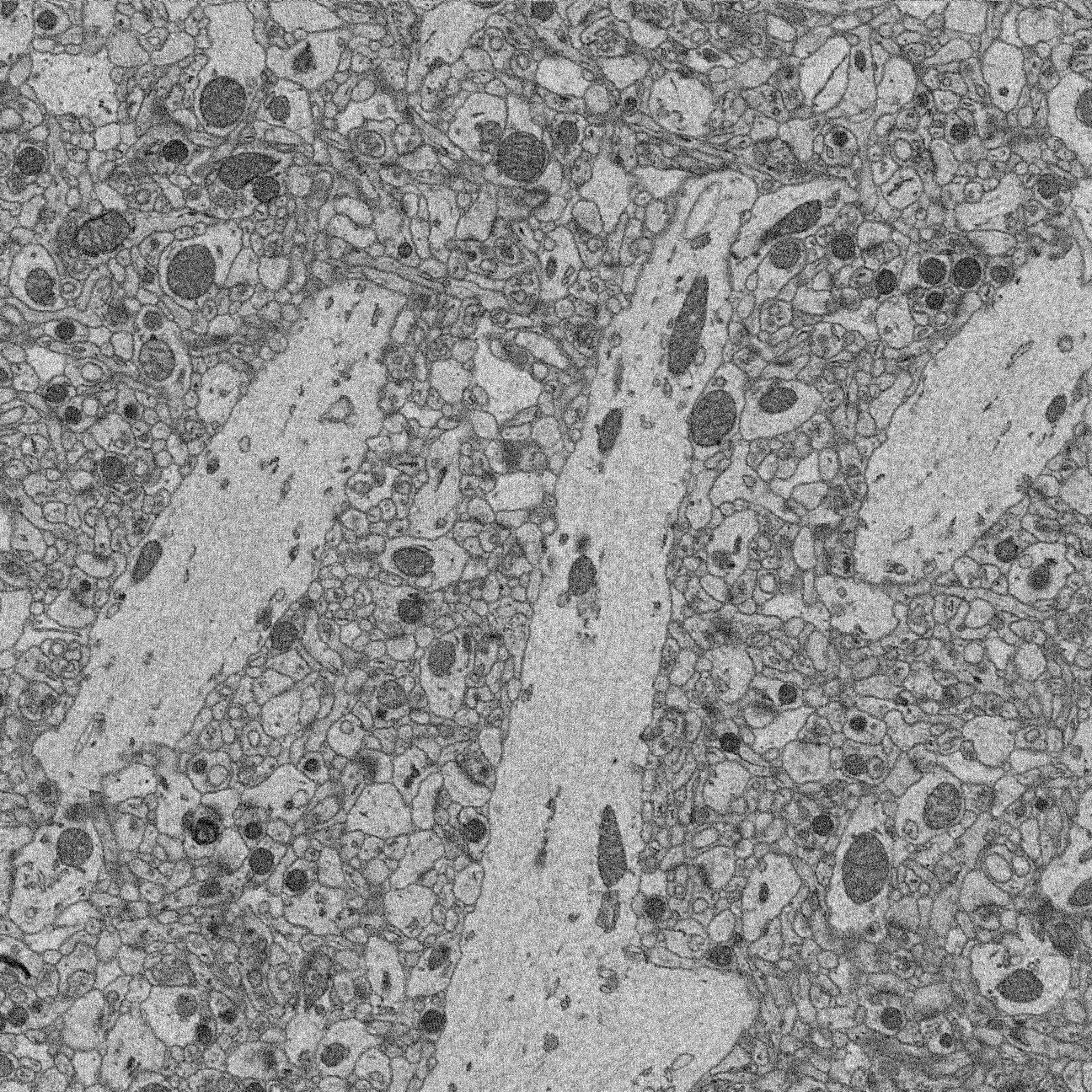

Supplement: Supplementary file 7 — Source data Fig. 5 [file 44321_2024_147_MOESM7_ESM.zip › Figure 5/5E/Figure 5 E.tif]

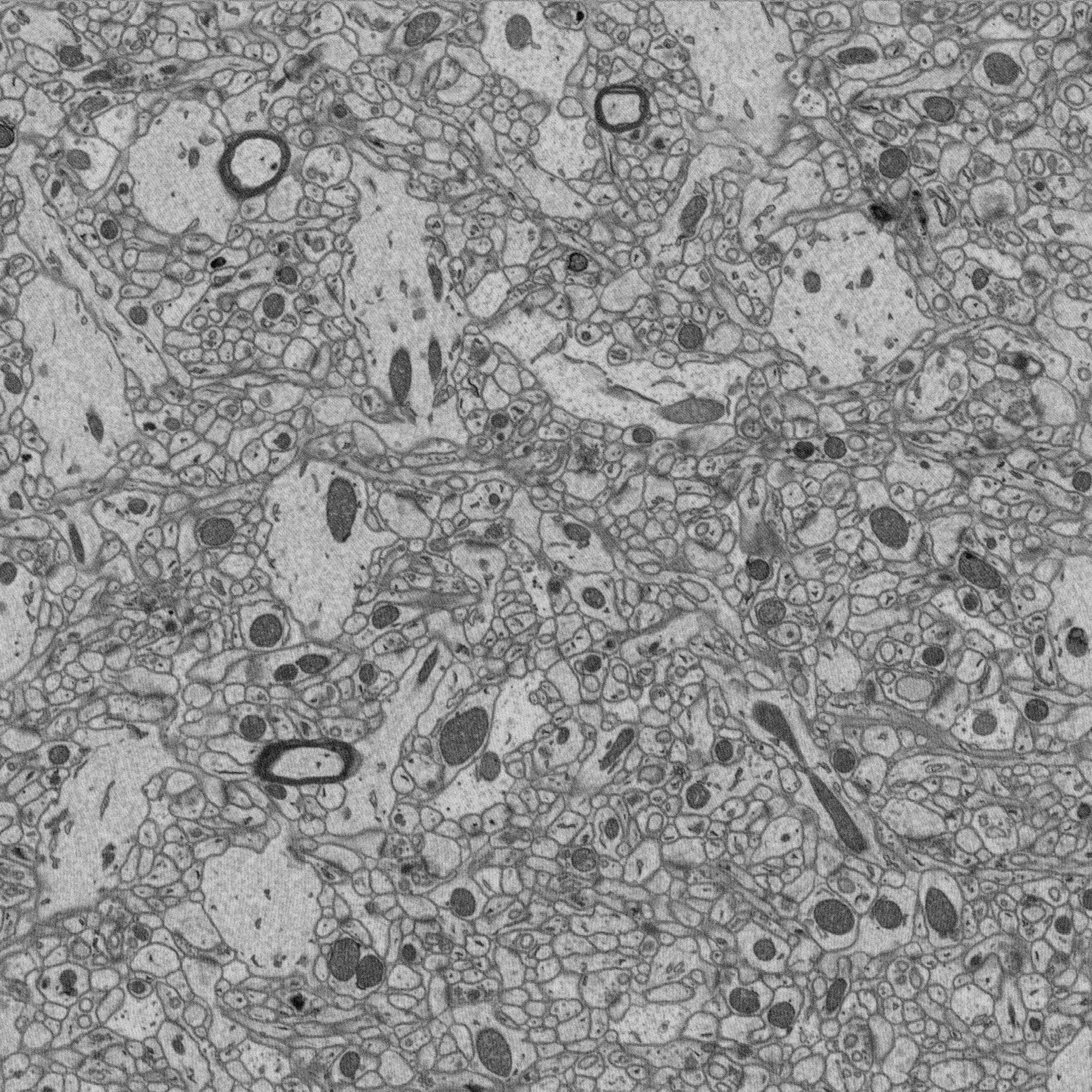

Supplement: Supplementary file 7 — Source data Fig. 5 [file 44321_2024_147_MOESM7_ESM.zip › Figure 5/5F/Figure 5 F.tif]
