# Supplementary material for: Mutation in the mitochondrial chaperone TRAP1 leads to autism with more severe symptoms in males
Source: EMBO Mol Med. 2024 Sep 27;16(11):2976–3004. doi: 10.1038/s44321-024-00147-6 (PMC11554806; doi:10.1038/s44321-024-00147-6)
Supplement: Supplementary file 8 — Expanded View Figures [file 44321_2024_147_MOESM8_ESM.pdf]

Expanded View Figures

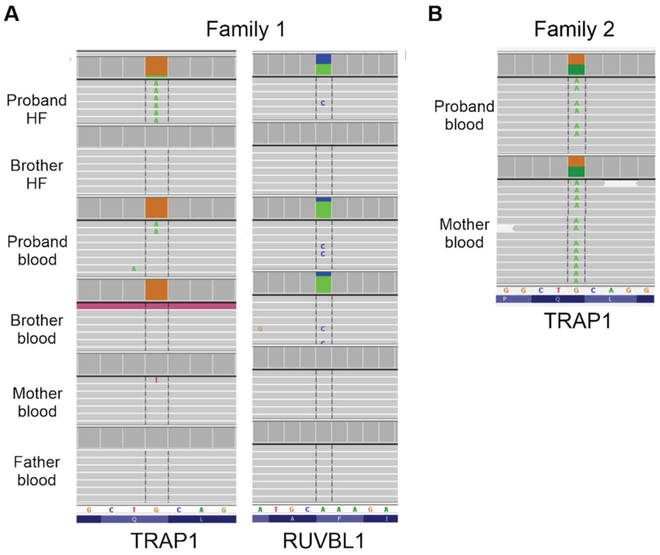

Figure EV1. NGS-based deep amplicon sequencing of postzygotic *TRAP1* and *RUVBL1* variants identified by whole-exome sequencing in a pair of ASD-discordant MZTs and the *TRAP1* p.Q639* variant from a replication cohort individual.

(**A**) Variants *TRAP1* p.Q639* and *RUVBL1* p.F329L were verified in the ASD-affected twin and his ASD-unaffected twin brother in DNA samples purified from hair follicles (HF) and blood; parental analysis was done on blood samples only. In the ASD-affected twin the VAF of *TRAP1* p.Q639* in HF DNA sample was 8% (genomic position coverage 13368×), and in the blood DNA sample, the VAF was 2% (genomic position coverage 27384x). In the unaffected brother HF DNA the variant was not present (genomic position coverage 7779x), while in the blood, the VAF was 2% (genomic position coverage 31972x). In the parent samples, only the wild-type sequence was identified (coverage 26714x for mother and 28222x for father). In the ASD-affected twin, the VAF of RUVBL1 p.F329L in HF DNA was 48% (coverage 54610×) and in the blood sample the VAF was 22% (coverage 49383×). In the unaffected brother's HF DNA only the wild-type sequence was identified (coverage 52237×), while in blood the VAF was 22% (coverage 40726×). In the parents, only the wild-type sequence was identified (coverage 57498× for mother and 51788× for father). (**B**) Verification of the heterozygous *TRAP1* p.Q639* variant in an ASD patient from the replication cohort revealed inheritance from a ASD-unaffected mother (VAF 50% in both proband and mother); DNA from the proband's father was not available for testing. Deep amplicon sequencing results were viewed with the Integrative Genomics Viewer (IGV) tool.

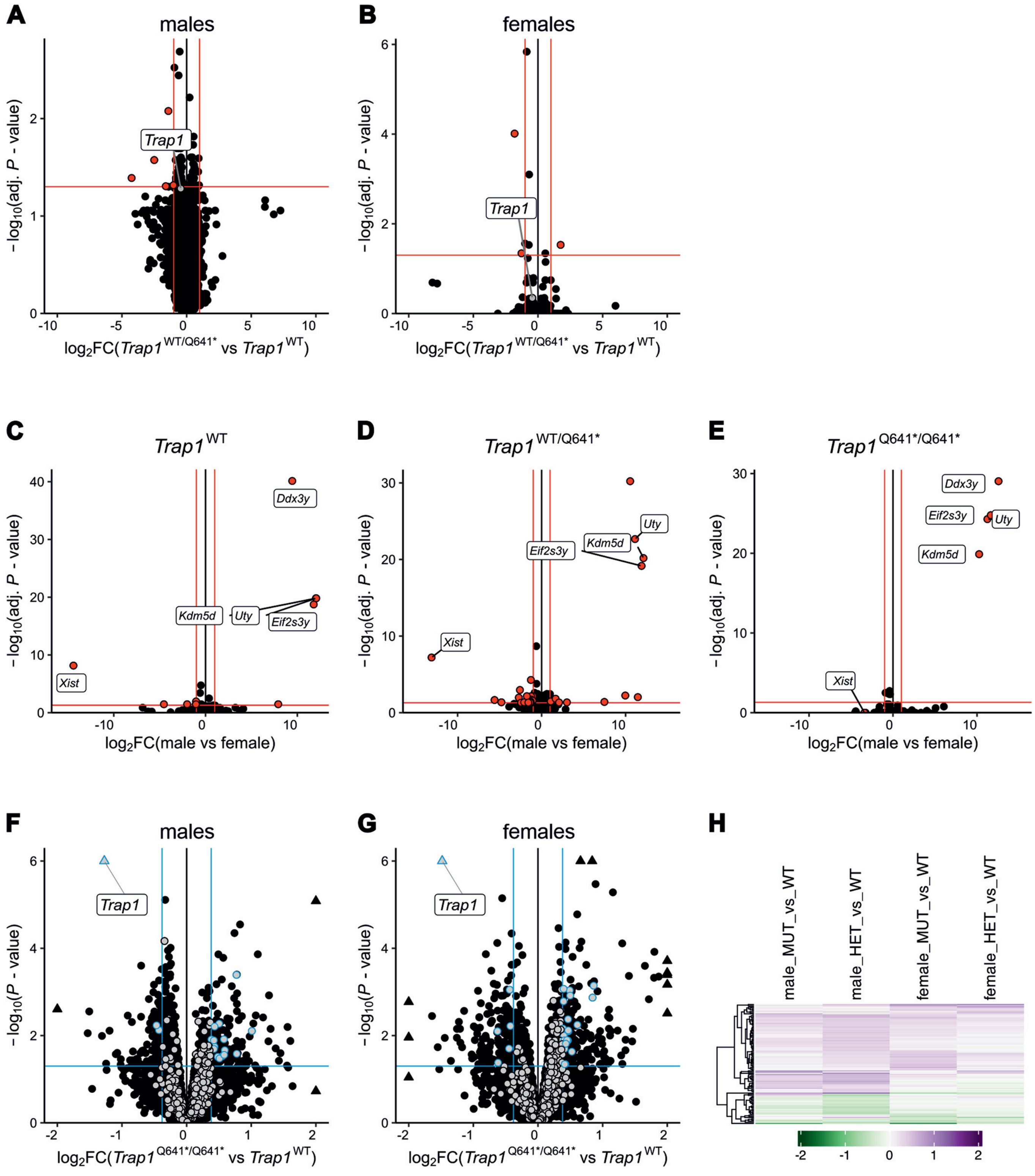
A
males
Trap1
-log10(adj. P - value)
log2FC(Trap1 WT/Q641* vs Trap1 WT)
B
females
Trap1
-log10(adj. P - value)
log2FC(Trap1 WT/Q641* vs Trap1 WT)
C
Trap1 WT
Ddx3y
Kdm5d
Uty
Eif2s3y
Xist
-log10(adj. P - value)
log2FC(male vs female)
D
Trap1 WT/Q641*
Uty
Kdm5d
Eif2s3y
Xist
-log10(adj. P - value)
log2FC(male vs female)
E
Trap1 Q641*/Q641*
Ddx3y
Eif2s3y
Uty
Kdm5d
Xist
-log10(adj. P - value)
log2FC(male vs female)
F
males
Trap1
-log10(P - value)
log2FC(Trap1 Q641*/Q641* vs Trap1 WT)
G
females
Trap1
-log10(P - value)
log2FC(Trap1 Q641*/Q641* vs Trap1 WT)
H
male_MUT_vs_WT
male_HET_vs_WT
female_MUT_vs_WT
female_HET_vs_WT
-2
-1
0
1
2

© The Author(s)

Figure EV2. Transcriptional changes in *Trap1$^{Q641*/Q641*}$*, *Trap1$^{WT/Q641*}$* and *Trap1^{WT}* mice.

(**A–G**) Volcano plots representing the global differential gene expression in RNA-Seq analysis of the hippocampi of *Trap1^{WT}*, *Trap1$^{WT/Q641*}$* and *Trap1$^{Q641*/Q641*}$* for male and female mice ($n = 3$–4 animals/group). (**A–E**) The x-axis indicates $\log_2$ fold changes ($\log_2$FC) of gene expression levels in *Trap1$^{WT/Q641*}$* versus *Trap1^{WT}* mice in males (**A**) and females (**B**) or changes in males versus females in *Trap1^{WT}* (**C**), *Trap1$^{WT/Q641*}$* (**D**) and *Trap1$^{Q641*/Q641*}$* (**E**). The *y*-axis indicates $-\log_{10}$ of adjusted *P* value (adj.*P* value). *P* values were calculated with Wald test statistics and were adjusted with Benjamini–Hochberg method. Black circles represent transcripts not differentially expressed, red circles represent transcripts significantly differentially expressed ($|\log2(FC)| > 1$, adj.*P* value < 0.05–thresholds designated by red lines on the plot). The topmost differential genes are labeled by gene symbols. (**F**, **G**) Volcano plots representing the global differential gene expression in RNA-Seq analysis of the hippocampi of Trap1^{WT} and Trap1$^{Q641*/Q641*}$ for male (**F**) and female (**G**) mice ($n = 3$–4 animals/group). The *x*-axis indicates log2 fold changes (log2FC) of gene expression levels in Trap1$^{Q641*/Q641*}$ versus Trap1^{WT} mice, and the *y*-axis indicates –log10 of *P* value (not the adj.*P* value). Black circles represent transcripts not differentially expressed, gray circles represent genes coding proteins with mitochondrial localization, circles with blue outlines represent transcripts significantly differentially expressed ($|\log2(FC)| > 0.38$, *P* value < 0.05–thresholds designated by blue lines on the plot). The triangle shapes on the plots are for the outlier transcripts that had values out of the scale presented on the plot and their values were displaced by the maximum/ minimum plotted value (changes applied on both axis). (**H**) Heatmap representation of expression changes of 103 genes coding proteins with mitochondrial localization on transcript levels from the RNA-Seq analysis. The genes were chosen if were significantly differentially expressed ($|\log2(FC)| > 0.38$, *P* value < 0.05) in any of the plotted comparisons. The colors represent $\log_2$ of fold changes. Green represents genes which expression was lower in *Trap1$^{Q641*/Q641*}$* or *Trap1$^{WT/Q641*}$* and higher in *Trap1^{WT}*, whereas violet represents the opposite.

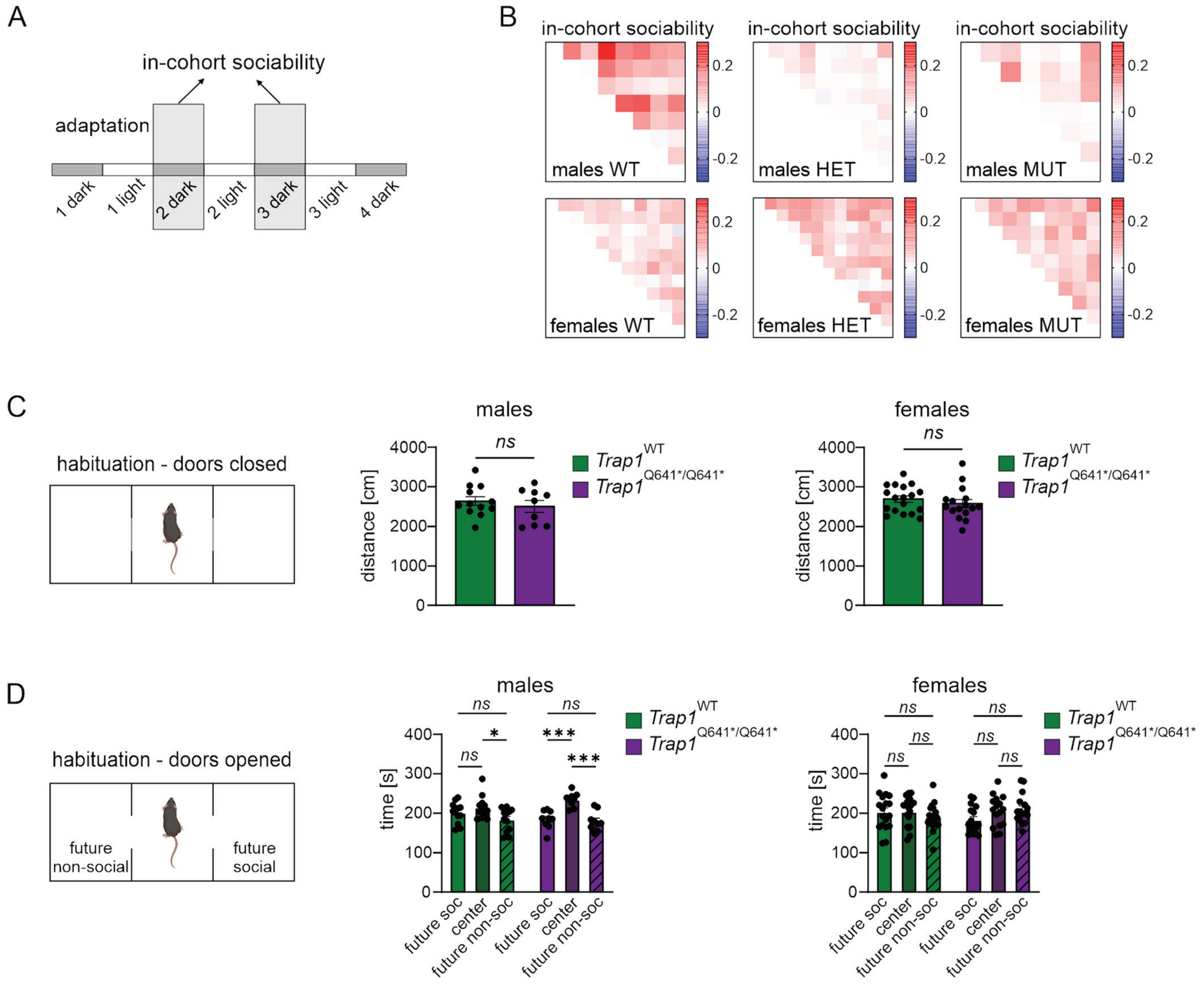

Figure EV3. Eco-HAB and three-chamber behavioral analysis of *Trap1*^{WT}, *Trap1*$^{WT/Q641*}$ and *Trap1*$^{Q641*/Q641*}$ mice.

(**A**, **B**) Eco-HAB behavioral analysis of *Trap1*^{WT}, *Trap1*$^{WT/Q641*}$ and *Trap1*$^{Q641*/Q641*}$ mouse cohorts. (**A**) Schematic depicting the experimental timeline and data used for analysis of Eco-HAB behavioral measures. Locomotor activity was assessed in 12-h bins and "in-cohort sociability" was calculated from the data collected during the 2nd and 3rd dark phase. (**B**) Heat maps depicting "in-cohort sociability" results. Each small square represents the "sociability" parameter for one pair of subjects, the color scale used is *blue* to *red*, with the *blue* representing "low sociability" and *red* "high sociability". $N = 7$–12 animals/group. Frequency distribution histograms for all pairs of animals from different cohorts are presented in main Fig. 3D,E. (**C**, **D**) Habituation phases of the three-chamber social approach task in *Trap1*^{WT} and *Trap1*$^{Q641*/Q641*}$ mice. (**C**) Scheme showing the first habituation phase—with closed doors (left panel). The graphs show distance traveled by each mouse in the center chamber during 10 min. No differences in the locomotor activity were observed between the genotypes neither in males nor in females ($P > 0.05$, Mann–Whitney test; error bars indicate SEM). (**D**) Scheme showing the second habituation phase—with doors opened (left panel). The graphs show time spent by a mouse in each of the chambers during 10 min. Male *Trap1*$^{Q641*/Q641*}$ mice preferred to spend more time in the already familiar center chamber as compared to the novel side chambers ($P < 0.0001$, two-way ANOVA, post hoc Tukey's multiple comparisons test; error bars indicate SEM). $N = 9$–12 and 16–18 animals/group for males and females respectively.

 © The Author(s)

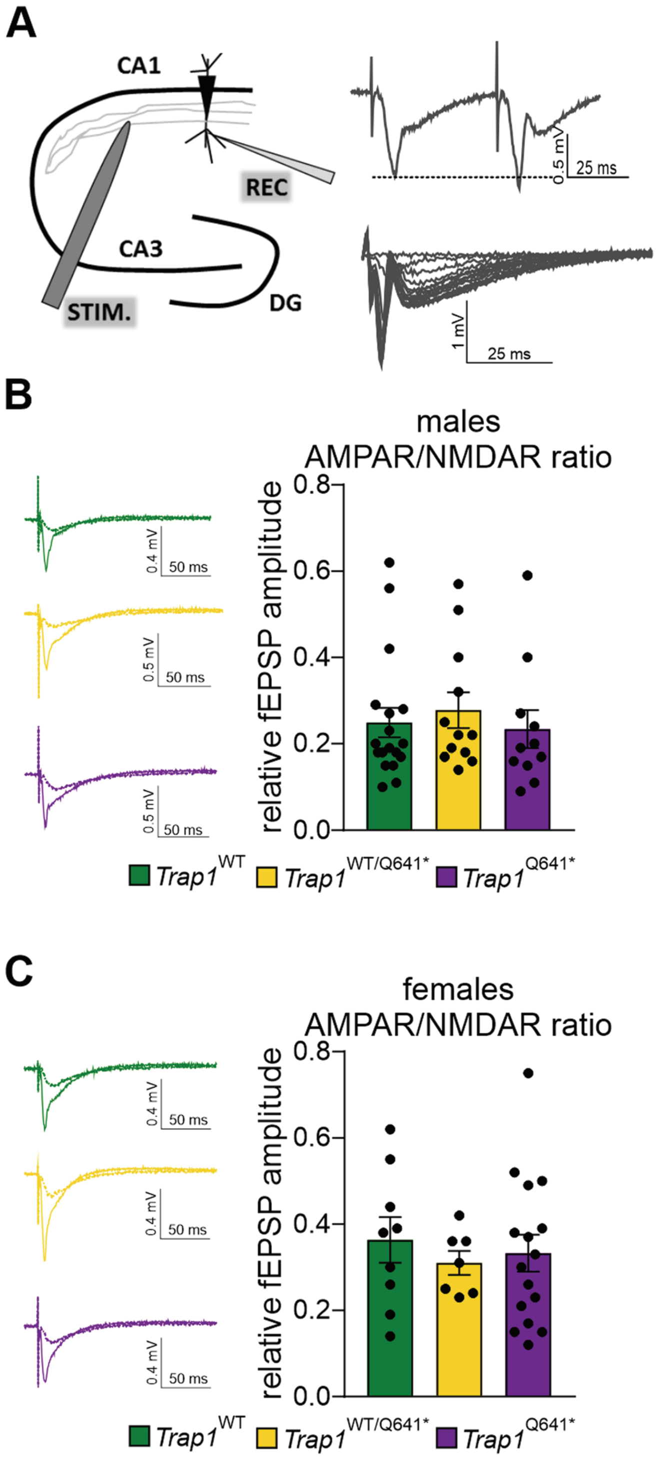

Figure EV4. Electrophysiological recordings of compound AMPAR- and NMDAR-mediated fEPSPs in the CA1 hippocampal region of *Trap1* mice.

(**A**) Schematic of the electrophysiological recording setup depicting positions of stimulating (STIM) and recording (REC) electrodes in the CA1 hippocampal region. Top right, example trace of fEPSPs scaling in response to paired stimulation of Schaffer collaterals (interstimulus interval 50 ms). Bottom right, example traces of compound fEPSPs recorded in response to monotonically increasing stimuli applied to Schaeffer collaterals. (**B**) Quantification of changes in fEPSP amplitude following the application of the AMPAR antagonist DNQX. (20 μM). Sensitivity to DNQX and thus AMPAR/NMDAR ratio was not significantly different among male groups (one-way ANOVA, $P > 0.05$; error bars indicate SEM). (**C**) Quantification of changes in fEPSP amplitude following the application of the AMPAR antagonist DNQX. Sensitivity to DNQX was not significantly different among female groups (one-way ANOVA, $P > 0.05$; error bars indicate SEM). Insets in (**B**, **C**) show example recordings of compound fEPSPs before and after DNQX application. $N = 3$–6 animals, $n = 12$–25 slices (males); $N = 3$–4 animals, $n = 12$–17 slices (females).

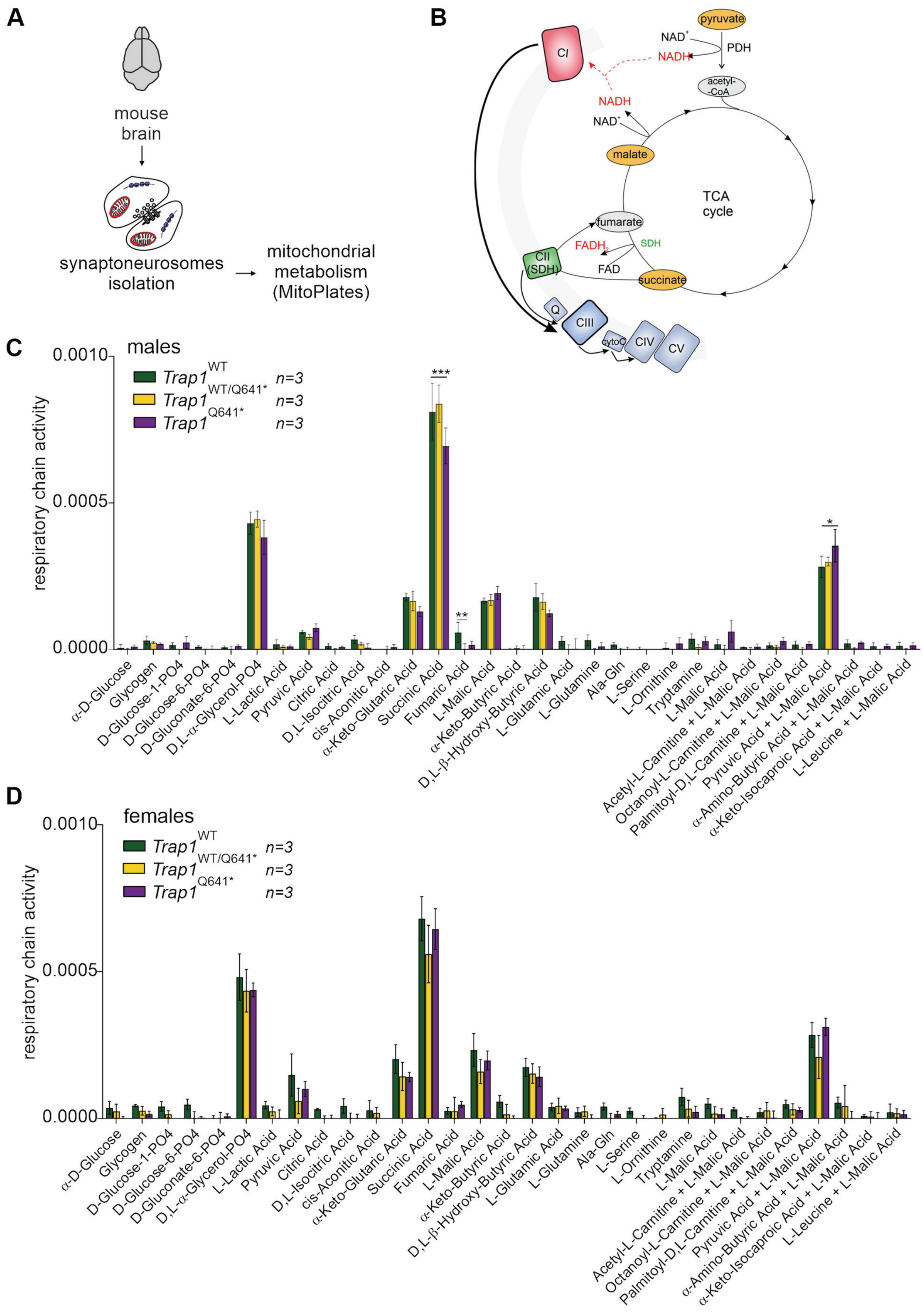
A
mouse
brain
synaptoneurosomes
isolation
mitochondrial
metabolism
(MitoPlates)
B
CI
pyruvate
NAD+
NADH
PDH
acetyl-CoA
NADH
NAD+
malate
TCA
cycle
fumarate
FADH2
SDH
CII
(SDH)
FAD
succinate
Q
CIII
cytoC
CIV
CV
C
males
Trap1WT n=3
Trap1WT/Q641* n=3
Trap1Q641* n=3
respiratory chain activity
0.0010
0.0005
0.0000
D
females
Trap1WT n=3
Trap1WT/Q641* n=3
Trap1Q641* n=3
respiratory chain activity
0.0010
0.0005
0.0000
α-D-Glucose
Glycogen
D-Glucose-1-PO4
D-Glucose-6-PO4
D-Gluconate-6-PO4
D,L-α-Glycerol-PO4
L-Lactic Acid
Pyruvic Acid
Citric Acid
D,L-Isocitric Acid
cis-Aconitic Acid
α-Keto-Glutaric Acid
Succinic Acid
Fumaric Acid
L-Malic Acid
α-Keto-Butyric Acid
D,L-β-Hydroxy-Butyric Acid
L-Glutamic Acid
L-Glutamine
Ala-Gln
L-Serine
L-Ornithine
Tryptamine
L-Malic Acid
Acetyl-L-Carnitine + L-Malic Acid
Octanoyl-L-Carnitine + L-Malic Acid
Palmitoyl-D,L-Carnitine + L-Malic Acid
Pyruvic Acid + L-Malic Acid
α-Amino-Butyric Acid + L-Malic Acid
α-Keto-Isocaproic Acid + L-Malic Acid
L-Leucine + L-Malic Acid

 © The Author(s)

Figure EV5. Functional mitochondrial phenotyping of synaptoneurosomes isolated from mouse brains (cortex and hippocampus) of male and female *Trap1* mice.

The electron flow rates in the electron transport chain from 31 different bioenergetic substrates, including glycolysis, TCA cycle intermediates, fatty acids and amino acids, were measured using MitoPlates™. (**A**) Scheme showing the experimental workflow. (**B**) Scheme depicting selected substrate supply for mitochondrial respiration. Substrates differentially utilized in *Trap1*$^{Q641*/Q641*}$ synaptoneurosomes are marked in *yellow*. (**C**) In male *Trap1*$^{Q641*/Q641*}$ mice decreased usage of succinate (***$P < 0.001$) and fumarate (**$P < 0.01$) was observed. Also, increased consumption of pyruvate +malate (*$P < 0.05$) was noticed. (**D**) In contrast, in females no differences in usage of mitochondrial energy substrates were observed. Results are presented as the average rate/min/µg of protein, +/− SEM ($n = 3$ per genotype/sex; two-way ANOVA, post hoc Sidak's multiple comparisons test).
